# Serial Passaging of Seasonal H3N2 Influenza A/Singapore/G2-31.1/2014 Virus in MDCK-SIAT1 Cells and Primary Chick Embryo Cells Generates HA D457G Mutation and Other Variants in HA, NA, PB1, PB1-F2, and NS1

**DOI:** 10.3390/ijms232012408

**Published:** 2022-10-17

**Authors:** Daryl Zheng Hao Aw, Keng Kai Heng, Jovian Yee Han Heok, Xin Yang Kong, Hui Chen, Tong Zhang, Weiwei Zhai, Vincent T. K. Chow

**Affiliations:** 1 NUHS Infectious Diseases Translational Research Program, Host and Pathogen Interactivity Laboratory, Department of Microbiology and Immunology, Yong Loo Lin School of Medicine, National University of Singapore, Singapore 117545, Singapore; 2 NUS High School of Mathematics and Science, Singapore 129957, Singapore; 3 Human Genetics, Genome Institute of Singapore, Agency for Science, Technology and Research, Singapore 138672, Singapore; 4 Key Laboratory of Zoological Systematics and Evolution, Institute of Zoology, Chinese Academy of Sciences, Beijing 100101, China

**Keywords:** H3N2 influenza, serial passaging, viral RNA quasispecies, mutations, MDCK-SIAT1 cells, chick embryo fibroblasts, vaccine efficacy, influenza virulence

## Abstract

Influenza remains one of the most prevalent viruses circulating amongst humans and has resulted in several pandemics. The prevention and control of H3N2 influenza is complicated by its propensity for evolution, which leads to vaccine mismatch and reduced vaccine efficacies. This study employed the strategy of serial passaging to compare the evolution of the human seasonal influenza strain A/Singapore/G2-31.1/2014(H3N2) in MDCK-SIAT1 versus primary chick embryo fibroblast (CEF) cells. Genetic analysis of the HA, NS1, NA, and PB1 gene segments by Sanger sequencing revealed the presence of specific mutations and a repertoire of viral quasispecies following serial passaging. Most quasispecies were also found in PB1, which exhibited consistently high transversion-to-transition ratios in all five MDCK-SIAT1 passages. Most notably, passage 5 virus harbored the D457G substitution in the HA2 subunit, while passage 3 virus acquired K53Q and Q69H mutations in PB1-F2. An A971 variant leading to a non-synonymous R316Q substitution in PB1 was also identified in MDCK-SIAT1 passages 2 and 4. With an increasing number of passages, the proportion of D457G mutations progressively increased and was associated with larger virus plaque sizes. However, microneutralization assays revealed no significant differences in the neutralizing antibody profiles of human-influenza-immune serum samples against pre-passaged virus and passage 5 virus. In contrast, viable virus was only detected in passage 1 of CEF cells, which gave rise to multiple viral RNA quasispecies. Our findings highlight that serial passaging is able to drive differential adaptation of H3N2 influenza in different host species and may alter viral virulence. More studies are warranted to elucidate the complex relationships between H3N2 virus evolution, viral virulence changes, and low vaccine efficacy.

## 1. Introduction

Influenza is one of the most prevalent viruses afflicting humans and has resulted in several pandemics over the past decades. The most common influenza subtypes that routinely circulate in the global population are the H1N1 and H3N2 subtypes. H3N2 has been associated with millions of deaths worldwide since its introduction in the 1968 pandemic [1,2]. Since then, the prevention and control of influenza has largely been reliant on influenza vaccines.

Currently, influenza vaccines are updated annually based on recommendations by the World Health Organization (WHO). However, vaccine efficacy against H3N2 has been shown to be lower than that against H1N1 and influenza B, although the specific underlying reasons remain unclear. Belongia and McLean [3] proposed a triad of factors that underpin low vaccine efficacy, including host immune responses, inherent virus-associated factors, and vaccine production processes.

Host immunity affecting vaccine efficacy has been attributed to original antigenic sin (OAS), in which antibody responses towards the influenza strain an individual is first exposed to are amplified with subsequent infections or vaccinations. This suggests the presence of an antigenic hierarchy in which immunologic memory towards the original infecting strain is strongest, often compromising immunity against later strains encountered by an individual [4,5,6]. This model is also supported by another study which demonstrated that later infections increase antibody titer levels against previously encountered influenza strains instead of the boosting antigen [7]. The association of the OAS model with low vaccine efficacy is further confounded by host factors, such as variations in age [8]. The exact causes and current understanding of the immune system’s roles in vaccine efficacy remain unclear, thus rendering its mitigation difficult.

In contrast, the production and manufacturing process of influenza vaccines (which are typically produced in embryonated chicken eggs) are more technical and may perhaps be an easier target to address low vaccine efficacy against influenza. The design and production of effective vaccines against H3N2 influenza remain challenging, largely due to the high viral evolution rate (termed antigenic drift), most commonly in the hemagglutinin (HA) gene which encodes the receptor-binding domain. After the prediction and selection of candidate vaccine viruses (CVVs) by WHO, the selected strains are expanded in embryonated eggs. However, the crux of the vaccine efficacy problem lies in this process, wherein egg adaptation of the CVVs occurs [9]. Egg adaptation arises due to differences in sialic acid (SA) receptors present in embryonated chicken eggs and humans. Recent circulating influenza strains are adapted to the SA receptors in the human upper respiratory tract, which are linked to galactose via alpha-2,6 linkages, as opposed to the alpha-2,3-linked SA receptors common in avian hosts and embryonated chicken eggs [10,11]. The propagation and passaging of these human-adapted influenza strains in embryonated chicken eggs drive a positive selection pressure on these strains to adapt to the avian receptors, culminating in antigenic drift. As a result of this deviation, the antibodies elicited by these CVVs tend to bind poorly to the contemporary H3N2 strains circulating in the human population [12]. Despite the drawbacks of egg-based vaccines, they remain the most common method of vaccine production due to the high virus yield, low costs, and established protocols [13,14].

Several studies reported mutations in H3N2 HA which confer increased receptor binding or reduced antibody-binding efficiencies to the virus [15,16,17]. In addition to HA, mutations have also been documented in other H3N2 influenza virus genes and proteins [18]. One study reported viral gene mutations following adaptation in mice, most notably a K615E substitution in the viral polymerase PA protein which was associated with lethality in vivo [19]. In addition, protective effects against antibody-mediated neutralization have been demonstrated in H3N2 virus possessing a glycosylation-associated neuraminidase (NA) mutation at amino acid residue 245 [20]. Studies on H3N2 virus evolution have identified the evolutionary significance of several codons contributing greatly to antigenic drift during egg adaptation [21,22,23].

There has been some emphasis on the development of cell-based influenza vaccines instead in order to ameliorate the effects of egg adaptation and antigenic drift. Influenza viruses are conventionally propagated in Madin–Darby canine kidney (MDCK) cells, which are suitable host cells for the generation of high-yield influenza vaccines [24,25]. Furthermore, influenza strains cultured in MDCK cells generally do not exhibit the same degree of antigenic drift observed in egg-passaged influenza strains. Park et al. [26] recorded no HA or NA mutations after serial passage of a H3N2 strain in a modified MDCK cell line, reflecting the relative genetic stability of influenza strains propagated in MDCK cells. This further strengthens its position as an alternative candidate over embryonated eggs for manufacturing influenza vaccines [27,28].

The MDCK-SIAT1 cell line is genetically engineered to exhibit higher expression of alpha-2,6-linked sialic acid receptors, and is superior for supporting the replication of recent human influenza viruses compared to conventional MDCK cells which express both alpha-2,6-linked and alpha-2,3-linked sialic acid receptors [29,30]. Similarly, whilst embryonated chicken eggs are traditionally used for influenza virus propagation, primary chick embryo fibroblast (CEF) cultures have also been increasingly employed, due to their greater ease of handling and management under experimental settings as compared to embryonated chicken eggs [31,32].

In this study, the passage adaptations of the seasonal influenza strain A/Singapore/G2-31.1/2014(H3N2) [33] in both MDCK-SIAT1 and primary CEF cells were explored and compared. The HA, NA, PB1, and non-structural protein 1 (NS1) genes of the passages were sequenced and analyzed for amino acid substitutions. The specific objectives were to study the effects of passage adaptation in driving H3N2 evolution in mammalian versus avian host cells, to investigate the repertoire of viral quasispecies arising from the accelerated evolutionary process, and to identify their possible association with low vaccine efficacy.

## 2. Results

### 2.1. Differential Infectivity of A/Singapore/G2-31.1/2014 in MDCK-SIAT1 and CEF Cells

**Figure 1:**
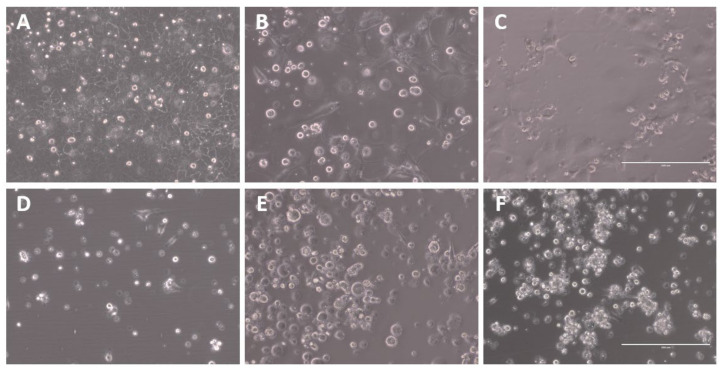
Representative images of viral CPE of A/Singapore/G2-31.1/2014 infection of MDCK-SIAT1 and CEF cells at 3 days post-infection (dpi). Microscopic images were captured using the EVOS XL microscope at 20× magnification. The scale bar (bottom right) represents 200 µm. (**A**) Uninfected MDCK-SIAT1 and infected MDCK-SIAT1 cells at (**B**) P1 and (**C**) P5. (**D**) Uninfected CEFs and infected CEFs at (**E**) P1 and (**F**) P5.

To evaluate the differential effects of passage adaptation in both human and avian cell lines, A/Singapore/G2-31.1/2014 was serially passaged in MDCK-SIAT1 and CEF cells for up to five passages, P1 to P5. Each passage was visually observed for the presence of viral CPE. CPE was evident for all MDCK-SIAT1 passages, as denoted by areas of clearing in the cell monolayer. However, typical CPE was not obvious in the CEF passages, although the cells appeared to be more clustered, likely as a result of their susceptibility to detach due to TPCK-trypsin (Figure 1).

**Figure 2:**
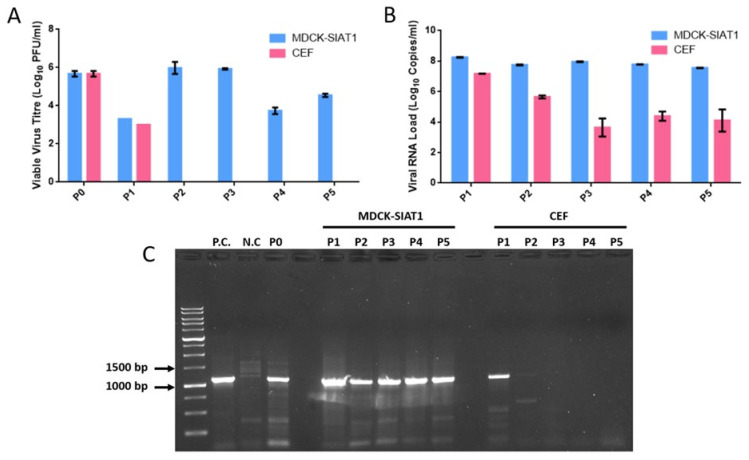
Viable A/Singapore/G2-31.1/2014 virus, total viral RNA quantification, and classical HA1 RT-PCR amplification of serial passage samples. (**A**) Live virus titers in the MDCK-SIAT1 and CEF passages at the day of harvest. Data represent mean values derived from duplicates. (**B**) Total viral RNA quantification in each MDCK-SIAT1 and CEF passage. Data represent mean values derived from triplicate analyses. (**C**) Screening of MDCK-SIAT1 and CEF passages for the detection of HA1 amplicons by classical RT-PCR and agarose gel electrophoresis. Influenza A/Aichi/2/1968(H3N2) and uninfected MDCK cells served as positive control (P.C.) and negative control (N.C.), respectively.

To verify the CPE observations, viable virus was detected by plaque assays of all MDCK-SIAT1 passages. Viable virus levels fluctuated in all MDCK-SIAT1 passages, with live virus load peaking at 5.98 log10 PFU/mL in P2. Although live virus load decreased from P3 to P4, it rebounded again in the final P5 passage to 4.54 log10 PFU/mL. In contrast, for the CEF passages, viable virus was only detected in P1 at 3 log10 PFU/mL (Figure 2A).

Conventional RT-PCR and real-time quantitative RT-PCR targeting viral HA1 and matrix genes were performed for further confirmation. Real-time RT-PCR analysis revealed similar trends to plaque assays, with consistently high viral RNA loads above 7 log10 copies/mL in MDCK-SIAT1 passages. On the other hand, viral RNA levels in CEF P2 to P5 were below 6 log10 copies/mL, likely reflecting the detection of non-viable virus fragments (Figure 2B). Viral HA1 was undetectable by conventional RT-PCR for CEF passages P2 to P5 (Figure 2C).

These findings indicate that A/Singapore/G2-31.1/2014 replicates efficiently in MDCK-SIAT1 cells, in comparison to CEFs, in which live virus could not be detected beyond a single passage. This highlights the lower capacity of A/Singapore/G2-31.1/2014 (a human virus isolate) in adapting to avian receptors in CEFs.

### 2.2. Specific Mutations of A/Singapore/G2-31.1/2014 Arise during Serial Passaging in MDCK-SIAT1 Cells

RT-PCR amplifications of full-length HA and NS1 genes were successful in all passage samples, except for CEF P2 to P5. Full-length NA and PB1 genes were also amplified successfully, except for all CEF passages. All amplicons were subjected to Sanger sequencing, and the resultant sequences were pairwise-aligned with the P0 sequences of the respective genes using the EMBL-EBI EMBOSS Needle tool. The sequencing results and specific mutations are summarized in Table 1.

Most notably, a non-synonymous amino acid substitution of D457G (aspartic acid to glycine) arising from a nucleotide replacement (A1399G) was identified in MDCK-SIAT1 P5. This substitution also corresponds to the D112G substitution based on the HA2 subunit nomenclature. In PB1 of P3, two non-synonymous amino acid substitutions of Q84P and G101C were detected. It is noteworthy that when the same nucleotide substitutions corresponding to Q84P and G101C were analyzed in the +1 open reading frame of the PB1 sequence that encodes the truncated protein PB1-F2, the resultant PB1-F2 substitutions were K53Q and Q69H. No specific amino acid substitutions were found in the NS1 and NA genes.

### 2.3. HA D457G Substitution Is Associated with Enhanced Viral Infectivity

**Figure 3:**
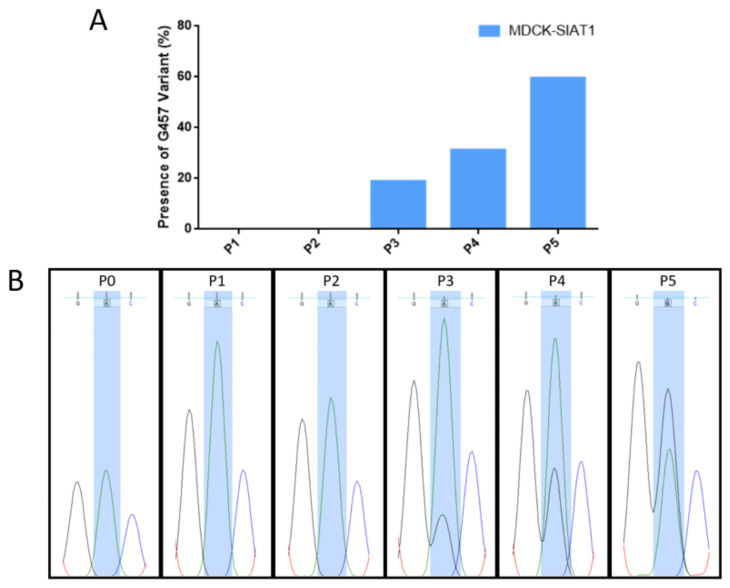
Presence or absence of the HA G457 variant (from the A1399G mutation) in different MDCK-SIAT1 passages. (**A**) Proportion of the height of the G nucleotide relative to the combined heights of both the G and A nucleotides. The height of each nucleotide peak was measured, and the proportion was expressed as a percentage. (**B**) Sequencing chromatograms for P0 and all MDCK-SIAT1 passages at nucleotide 1399. Chromatograms were visualized using FinchTV software.

Although the D457G substitution was dominant in P5 of the MDCK-SIAT1 passages, the G457 variant was initially observed in P3 as viral RNA quasispecies. There was a steady increase in the relative proportion of the G457 variant from P3 to P5 (Figure 3A). In P5, G457 replaced D457 as the dominant variant (Figure 3B), thus reflecting its evolutionary fitness over the wild-type D457.

**Figure 4:**
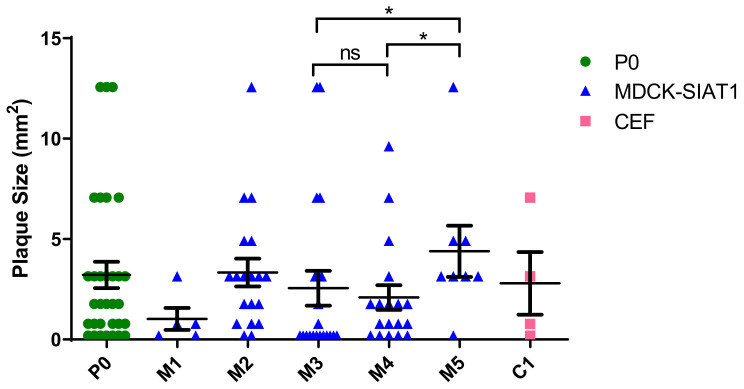
Mean virus plaque sizes (calculated as plaque areas) observed at each passage. The data represent mean values derived from virus plaque assays performed in duplicate. The unpaired Mann–Whitney U test was used for statistical analysis. * denotes *p* < 0.05 (significant); ns: non-significant. More details on the viral plaque sizes of the various virus passages are available in Supplementary File S1.

**Figure 5:**
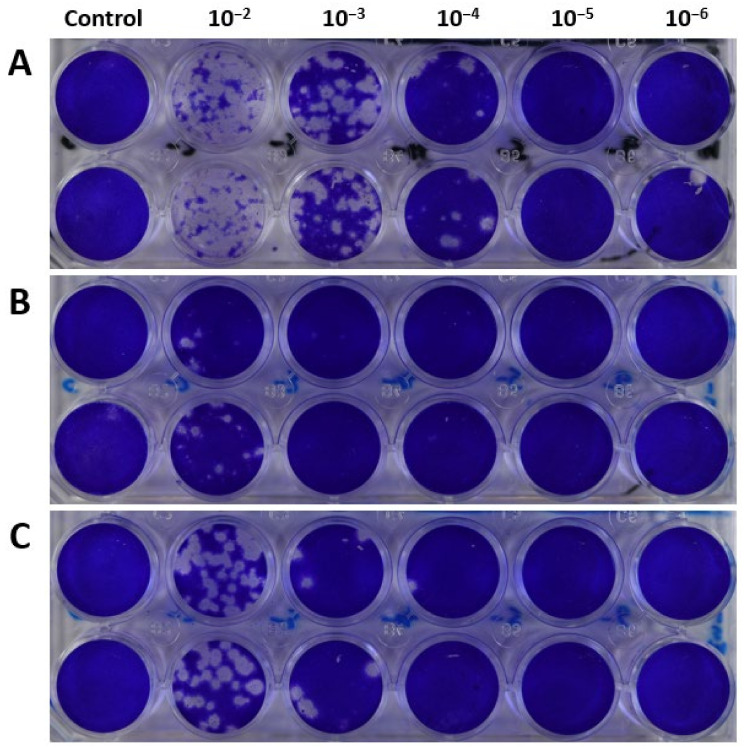
Representative virus plaque assay plates of the MDCK-SIAT1 passages (**A**) P3, (**B**) P4, and (**C**) P5, illustrating increases in plaque sizes from P3 to P5.

In tandem with the rise of the G457 variant within the viral population, virus plaque sizes also increased steadily from P3 to P5 (Figure 4 and Figure 5). Although the viable virus titer declined from P3 to P4, it rose to 4.54 log10 PFU/mL in P5 (Figure 2A), coinciding with G457 becoming the dominant variant in P5. Moreover, the mean plaque size in P5 was significantly greater than that of P3 and P4, thus implying an association between the evolutionarily fit D457G substitution with the heightened infectivity of P5 virus in MDCK-SIAT1 cells.

### 2.4. Viral RNA Quasispecies Are Numerous in Serially Passaged H3N2 Influenza Virus

The sequencing data were further analyzed for the presence of viral RNA quasispecies in all four genes, according to the criteria described in Section 4.8. For CEF P1, a total of 67 quasispecies leading to amino acid substitutions were identified in the HA sequence chromatograms, compared with 30 found in NS1. Within the HA gene, CEF P1 exhibited a higher number of transitions and transversions compared with MDCK-SIAT1 passages. In CEF P1, transitions and transversions detected within the NS1 gene were only approximately half of those of the HA gene.

Among MDCK-SIAT1 passages, the HA gene harbored the least number of quasispecies, compared to NS1, NA, and PB1. For both the NS1 and NA genes, there was a sharp increase in the number of non-synonymous mutations from MDCK-SIAT1 P2 to P3, before decreasing in P4. This corresponded with the 2.19 log10 PFU/mL reduction in live viral titer from MDCK-SIAT1 P3 to P4, suggesting a link between the non-synonymous substitutions from viral quasispecies and viral infectivity.

**Figure 6:**
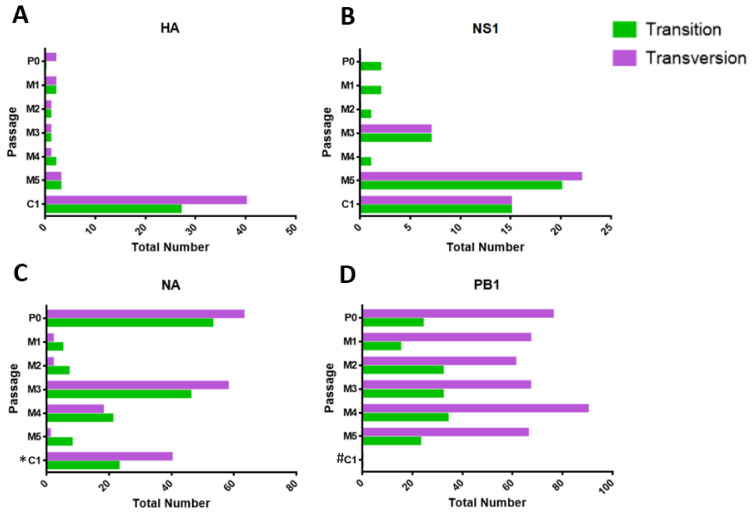
Numbers of transversions and transitions present in P0, MDCK-SIAT1 P1 to P5 (M1 to M5), and CEF P1 (C1) passages for viral genes (**A**) HA, (**B**) NS1, (**C**) NA, and (**D**) PB1. * indicates the number of transversions and transitions in CEF P1 that were only derived from partial NA genes (as full-length NA could not be amplified for this passage). # denotes that the numbers of transversions and transitions were not determined (due to the low quality of sequencing chromatograms for the sample). More details on the viral RNA quasispecies of the various virus passages are available in Supplementary File S1.

The PB1 gene exhibited the greatest number of RNA quasispecies, i.e., a total of 426 quasispecies culminating in 284 non-synonymous and 142 synonymous substitutions. In contrast to HA, NS1, and NA, the numbers of transversions in PB1 were consistently higher than the numbers of transitions in all MDCK-SIAT1 passages (Figure 6). One noteworthy variant of the PB1 gene detected in MDCK-SIAT1 P2 and P4 was A971, which gives rise to the non-synonymous substitution R316Q, where arginine is replaced by glutamine. Whilst this variant was absent in MDCK-SIAT1 P3, its re-emergence in MDCK-SIAT1 P4 after one passage implies its fitness.

### 2.5. Homology Models of HA and PB1 Mutant Proteins Predict Their Structural Alterations

**Figure 7:**
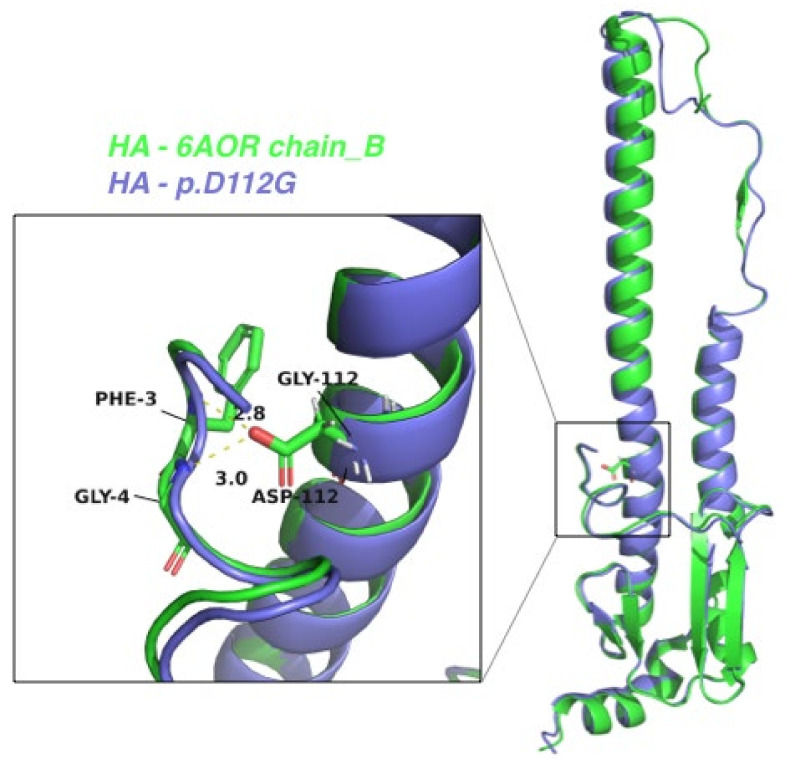
The superimposed structures between the HA protein of A/Brisbane/10/2007 (green) and the HA2 variant harboring the D112G mutation. A magnified image of the residue 112 region is depicted in the box. The residue 112 and its nearby residues are labeled, together with the distances in Angstrom (Å) units.

To predict the structural characterization of the mutations within H3N2 HA and PB1 proteins, we conducted homology modeling based on the published experimental structures. By treating chain B of the crystal structure of A/Brisbane/10/2007(H3N2) virus HA in apo form (PDB code: 6AOR) as the template structure of the HA protein [34], we modeled another HA structure whose sequence differed only in the D112G mutation (Figure 7). Detailed investigation revealed that D112 of HA interacts with the N-terminus of HA chain B, but this interaction would be lost in the D112G mutant (Figure 7). In view of the enhanced viral infectivity associated with this mutation, it is hypothesized that the HA2 N-terminus may play important roles in viral infective capability. Beyond the N-terminus, the detailed mechanisms by which HA2 influences viral virulence merit further exploration. Such investigations may offer new insights for designing HA2-based vaccine strategies.

**Figure 8:**
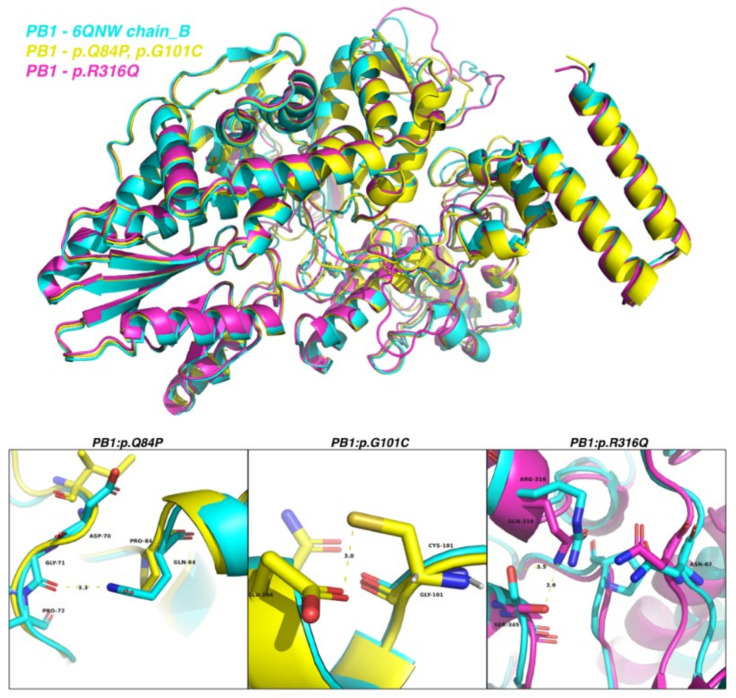
The superimposed structures of the PB1 protein of A/Northern Territory/60/1968 (cyan), PB1 with the Q84P and G101C mutations (yellow), and PB1 with the R316Q mutation (magenta). Magnified views of residues 84, 101, and 316 are depicted in the lower panel. Residues 84, 101, and 316 and their nearby residues are labeled in black, together with the distances in Angstrom (Å) units.

We adopted chain X of the crystal structure of A/Northern Territory/60/1968(H3N2) virus RNA-directed RNA polymerase catalytic subunit in apo state (PDB code: 6QNW) as the reference structure of the PB1 protein [35]. Homology modeling of PB1 predicted that Q84H would cause loss of interaction between Q84 and G71, whereas G101C would facilitate interaction between C101 and E104 (Figure 8). Given that PB1 plays a pivotal role in virus replication, further validation experiments are warranted to examine the impact of these two PB1 mutations on viral replication and virulence.

### 2.6. D457G Substitution in HA Does Not Alter the Susceptibility of A/Singapore/G2-31.1/2014 to Human Neutralizing Antibodies

**Figure 9:**
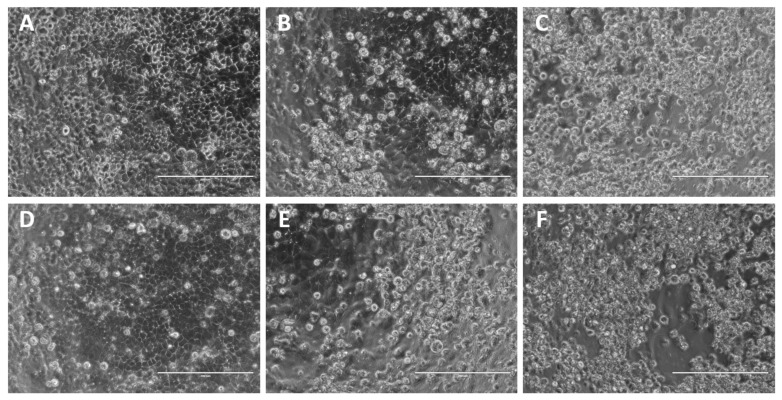
Representative images showing viral CPE of A/Singapore/G2-31.1/2014 in the microneutralization assays at 3 dpi. Images depict cells tested with serum samples FMK041 (**top row**) and FMT486 (**bottom row**). Microscopic images were captured using the EVOS XL microscope at 20× magnification. The scale bar (**bottom right**) represents 200 µm. “Serum-only” controls are shown in (**A**,**D**). MDCK-SIAT1 cells were infected with either P0 viral supernatant (**B**,**E**) or P5 viral supernatant (**C**,**F**).

The viral supernatants derived from MDCK-SIAT1 P0 (D457 variant) and P5 (G457 variant) were evaluated by neutralization tests (NTs) using a panel of 20 serum samples of known influenza-immune subjects to determine any differences between their antibody-binding profiles. There was no difference in NT results between P0 and P5 viruses for 16 serum samples, with confluent cell monolayers maintained either at neutralizing antibody titers of 1:160 or 1:320 (Table 2). In the remaining four serum samples, the neutralizing antibody titers against P5 virus were two-fold greater than those against P0 virus (albeit not a statistically significant difference). One study showed that a neutralizing antibody titer of 1:42 is associated with 50% protection against PCR-confirmed seasonal H3N2 infection [36]. Figure 9 shows representative CPE images from the neutralization assays. At serum dilutions ≥ 1:320 at 3 dpi, the CPE was evidently stronger for P5 virus (with almost total destruction of the cell monolayer), compared to the CPEs in the equivalent wells with P0 infection. Cells infected with P0 virus only displayed early CPE at the same time-point. These observations were consistent throughout the NT results for all 20 sera.

## 3. Discussion

The objectives of this study were to evaluate the impact of passage adaptation on H3N2 influenza evolution and to compare the differences between adaptation to both human and avian receptors. The repertoire of the resultant viral RNA quasispecies was also examined.

No viable virus was detected in the CEF passages beyond P1, suggesting low infectivity and viral replication. Sanger sequencing of the HA gene revealed a total of 67 transitions and transversions in CEF P1 alone. In stark contrast, only 17 HA transitions and transversions were identified in MDCK-SIAT1-passaged viruses after 5 passages. We hypothesize that this is most likely due to the incompatibility between the CEF alpha-2,3 N-linked SA receptors and the HA of A/Singapore/G2-31.1/2014, which is of human origin. This may have resulted in the greater number of adaptive mutations of the virus to compensate for the incompatible binding. Some studies have reported high yields of influenza virus in immortalized chick embryo cell lines, which are traditionally used for culturing avian influenza strains [37,38]. However, studies involving culture of clinical influenza virus isolates in CEFs remain limited. Another factor that may contribute to the poorer replication of A/Singapore/G2-31.1/2014 in CEFs is the inefficient release of viral progeny attributed to possible neuraminidase inhibition [39].

On the other hand, the MDCK-SIAT1 cell line was capable of supporting the propagation of A/Singapore/G2-31.1/2014 for up to five passages. Viable H3N2 virus was detected in all MDCK-SIAT1 passages by virus plaque assays and was further confirmed by real-time RT-PCR and Sanger sequencing. This is congruent with another study which documented that influenza H3N2 strains can successfully replicate to high viral titers in this cell line [29]. Another study found that MDCK-SIAT1 cells do not support H3N2 replication as well as conventional MDCK cells, although comparable yields of H1N1 can be obtained from both cell lines [30]. In our study, only one HA mutation was identified in MDCK-SIAT P5, but none were found in NS1. This trend is consistent with many studies that have shown minimal passage adaptation in conventional or modified MDCK cell lines. The high yield and relative genetic stability of the MDCK-SIAT1 passage isolates further reiterate the advantages of cell-based influenza vaccines over conventional egg-based vaccines [26,40,41].

In this study, we focused only on the HA, NS1, NA, and PB1 genes to examine their evolutionary patterns during serial passaging. HA and NA genes were selected to represent highly mutable genes in H3N2 influenza and because of their distinct roles in receptor binding and virus release. In contrast, NS1 and PB1 genes are genetically more stable and are involved in immune suppression and viral replication. No amino acid substitutions were found in NS1, which is unsurprising in view of its relatively high tolerance to mutation [42]. Interestingly, there were also no amino acid substitutions detected in NA.

In MDCK-SIAT1 P5, it is noteworthy that the D457G substitution is within the HA2 stalk domain. Strikingly, no mutations were observed within the HA1 region where the receptor-binding site (RBS) resides. This finding is interesting given that influenza mutations are prevalent within the RBS (and its surrounding regions), which forms the basis for explaining how HA-receptor incompatibility drives passage adaptation [21,22,43].

Our findings are similar to those of a previous study which documented a non-synonymous amino acid substitution at the same HA position, where aspartic acid was replaced by asparagine [44]. This HA mutation at amino acid 457 adds a new glycosylation site at the base of the stalk region, which interferes with trimerization and assembly of HA when present with other mutations, implicating HA transport from the rough endoplasmic reticulum to the Golgi apparatus. In our study, it was found that the D457G substitution is also likely to affect the structure of the stalk region due to the drastic alteration from negatively charged aspartic acid to non-polar and uncharged glycine (Figure 7).

Compared to preceding passages, the D457G HA mutation (also designated as D112G in HA2 nomenclature) was associated with enhanced infectivity, as was evident from the higher viral titers and larger plaque sizes, implying the role of HA2 in influenza virulence. In addition, the stronger CPEs observed in cells infected with P5 viral supernatant may also be attributed to the repertoire of quasispecies in other genes that may have conferred augmented virulence. This observation regarding CPE further supports that A/Singapore/G2-31.1/2014 acquired increasing virulence following serial passaging in MDCK-SIAT1 cells. The D457G HA mutation is known to result in elevated fusion pH and decreased stability of HA [45]. Influenza HA is typically cleaved into the HA1 and HA2 subunits, with the latter mediating viral uncoating and fusion. Despite HA2 being conserved, mutations do occur within the HA2 region, albeit to a lesser extent than in HA1 [46,47]. Its immunogenicity coupled with its highly conserved nature render HA2 a suitable candidate target for eliciting broadly inhibitory antibodies [48,49,50].

We also noted an increase in transitions and transversions of NA quasispecies at MDCK-SIAT1 passage 3, which diminished by passage 5. It is interesting that this was inversely correlated with the rise of the G457 HA variant and increase in virus plaque size from P3 to P5.

Interestingly, the greatest number of viral quasispecies was observed in PB1, which correlates with its high number of transversions over transitions in every MDCK-SIAT1 passage (Figure 6D). Specifically, the R316Q mutation in PB1 was detected in passages 2 and 4. Furthermore, non-synonymous amino acid substitutions, i.e., K53Q (lysine to glutamine) and Q69H (glutamine to histidine), were identified in the truncated PB1-F2 protein of passage 3. To the best of our knowledge, there have been no previous reports of the K53Q mutation in H3N2 influenza. However, residue 69 of PB1-F2 has been characterized previously.

PB1-F2 downregulates interferon production [51] and upregulates viral RNA polymerase activity via direct interaction with viral PB1 protein [52]. Amino acids 68 to 71 of PB1-F2 play important roles in PB1-F2 protein stability, subcellular localization, and interferon deregulation. Substitutions of T68I, Q69L, D70V, and S71F in H1N1 PR8 virus promote PB1-F2 protein half-life and stability, mitochondrial localization, and interferon antagonism [53]. Moreover, the unique I68, L69, and V70 motif in PB1-F2 of PR8 mediates cytotoxicity and predisposes to secondary pneumococcal pneumonia [54]. PB1-F2 inhibits the induction of type I interferon at the level of the mitochondrial antiviral signaling (MAVS) adaptor protein [55]. The C-terminal portion of PB1-F2 (amino acids 38 to 87) binds to MAVS and mediates interferon antagonist activity. The PB1-F2 N66S mutant binds more efficiently to MAVS, thus inhibiting MAVS-mediated interferon synthesis by reducing the mitochondrial membrane potential [51]. In our study, the non-synonymous Q69H substitution is similar to that of Q69L. We hypothesize that Q69H may confer properties on PB1-F2 similar to those of Q69L. Interestingly, the PB1 protein of H7N9 influenza virus can also counteract the innate immune response by targeting MAVS for selective autophagic degradation mediated by NBR1 [56].

The microneutralization results demonstrated few differences in the ability of human serum samples to bind and neutralize A/Singapore/G2-31.1/2014, even after a series of passages and the generation of multiple genetic variants within different influenza genes. No difference in neutralizing antibody titers was found between MDCK-SIAT1 P5 and P0 viruses in 80% (16/20) of the serum samples. However, the P5 virus was slightly more susceptible than the P0 virus to neutralization in 20% (4/20) of the tested sera. Given that D457G resides within the HA2 subunit, which mediates fusion of the viral envelope with the host endosomal membrane, it is likely that this mutation has negligible impact on the HA receptor-binding site. Despite exhibiting similar neutralizing antibody titers, the CPE observed upon infection of cell monolayers with P5 virus was evidently stronger than that with the P0 parental virus.

Although our results indicate that increased H3N2 influenza infectivity may be attributed to the HA2 D457G and PB1-F2 mutations in MDCK-SIAT1 P5 and P3, respectively, they do not preclude potential contributions from other influenza gene segments that were not sequenced. Influenza evolution and virulence can be directed by more than one mutation due to epistatic interactions across several other influenza genes, such as NA and nucleoprotein [16,57,58].

This study has certain limitations, e.g., only five serial virus passages were investigated and sequencing of the entire viral genome was not performed. Variants or mutations may be present in other H3N2 influenza genes which may explain our observations. In addition, the serum samples tested in the microneutralization assays only represent the in vitro effects of human neutralizing antibodies against the passaged virus [59].

Therefore, more detailed studies are warranted to elucidate the molecular mechanisms underpinning influenza virus adaptation and evolution. It is crucial to carry out sequencing of all H3N2 influenza genes of the passaged viruses to unravel other mutations of biological importance [60]. Future studies should also compare the results of virus passaging in embryonated eggs versus CEF cultures. In vivo experiments are also vital to gain better insights into the virulence and effects of serially passaged influenza viruses in animal models. These future investigations can deepen our understanding of the complex interactions between viral quasispecies and epistatic interactions. Such studies can also help to mitigate the undesirable effects of egg adaptation and improve the design of influenza vaccines.

## 4. Materials and Methods

### 4.1. Establishment of CEF Cultures

CEF cultures were established from 11-day-old specific-pathogen-free embryonated chicken eggs, as previously described [61], with some minor modifications. Briefly, each chick embryo was extricated, and isolated body tissue was homogenized in 1× trypsin before being filtered through a 40 µm cell strainer. Cells were collected by centrifugation at 580× *g* for 10 min at 20 °C and resuspended in 10 mL of Eagle’s minimum essential medium or EMEM (ATCC, Manassas, VA, USA) with 10% fetal bovine serum (FBS). Cells (10^6^) were propagated in EMEM (with 10% FBS) containing 1× antibiotic–antimycotic solution (A5955, Sigma-Aldrich, St. Louis, MO, USA) at 37 °C with 5% CO_2_. For subsequent passages, established cultures were maintained in EMEM free of antibiotic–antimycotic solution under the same conditions. This protocol was approved by the Institutional Animal Care and Use Committee of the National University of Singapore.

### 4.2. Cells and Virus

MDCK-SIAT1 cells were propagated in Dulbecco’s modified Eagle’s medium or DMEM (Thermo Fisher Scientific, Waltham, MA, USA) supplemented with 10% FBS and 1 mg/mL G418 sulfate (Promega, Madison, WI, USA). Conventional MDCK cells were maintained in EMEM supplemented with 10% FBS. Both cell lines were maintained at 37 °C with 5% CO_2_. Live culture of A/Singapore/G2-31.1/2014(H3N2) virus was initiated in MDCK-SIAT1 cells and maintained in serum-free DMEM at 35 °C with 5% CO_2_. The initial harvested viral culture supernatants were designated as passage zero (P0).

### 4.3. Serial Passaging of A/Singapore/G2-31.1/2014(H3N2) Virus

A total of five passages were carried out. Sub-confluent monolayers of MDCK-SIAT1 and CEF cells (seeded at 10^5^ cells the previous day) were infected with 50 µL of P0 in triplicate. Cells were incubated at 35 °C for 1 h before adding 1 mL of DMEM (for MDCK-SIAT1 cells) or EMEM (for CEFs) containing 0.5 µg/mL TPCK-trypsin—this was designated passage 1 (P1). Cells were then further incubated at 35 °C for up to 72 h. On the day of harvest, the well exhibiting the strongest cytopathic effect (CPE) was harvested. The harvested culture (150 µL) was diluted two-fold with either DMEM or EMEM supplemented with 1× antibiotic–antimycotic and 0.5 µg/mL TPCK-trypsin. The diluted virus culture (100 µL) was then used to initiate P2, as described above. Subsequent passages were performed in the same manner until P5. For all mock-infected controls, DMEM or EMEM was used without virus.

### 4.4. Live Virus Quantification by Plaque Assays

The quantity of viable viruses in each passage supernatant and their infectivity were determined by virus plaque assays. Sub-confluent MDCK-SIAT1 cells were infected in duplicate with ten-fold dilutions of the harvested passage supernatants for 1 h. Thereafter, the inoculum was removed and cells were overlaid with 1.2% Avicel BioPolymer (FMC, Philadelphia, PA, USA) and further incubated for 72 h. The cells were then fixed with 4% paraformaldehyde in phosphate-buffered saline (PBS) for 1 h and stained with 1% crystal violet solution for 15 min for plaque visualization and counting. The diameter of each plaque was also measured and the mean area calculated [62].

### 4.5. RNA Extraction and Screening by HA1 RT-PCR Amplification

Viral RNA was extracted from each virus passage using the QIAamp viral RNA mini kit (Qiagen, Hilden, Germany), according to the manufacturer’s protocol. The extracted RNA was quantified by a Nanodrop spectrophotometer, and 1 µg of viral RNA was used for cDNA synthesis using MMLV reverse transcriptase (Promega). The HA1 region of harvested virus from each passage was amplified via polymerase chain reaction (PCR) using GoTaq Green master mix (Promega) to screen for the presence of virus. PCR was initiated with a denaturation step of 95 °C for 2 min; followed by 34 thermal cycles each consisting of 45 s at 95 °C, 30 s at 44 °C, 45 s at 72 °C; with a final extension for 5 min at 72 °C. RT-PCR products were visualized via 1% agarose gel electrophoresis. The HA1 gene primers are shown in Table 3.

### 4.6. Real-Time Quantitative PCR to Determine Viral Load

Real-time quantitative PCR was carried out using primers specific to the influenza virus matrix gene to confirm the presence of the virus and to determine the viral load present in each passage. Each real-time PCR reaction was performed in triplicate using FastStart Essential DNA Green Master (Roche Molecular Systems, Pleasanton, CA, USA). Absolute viral RNA quantification of each passage was facilitated by including the standard curve constructed using P0 cDNA. The thermal cycling conditions were: 95 °C for 600 s; followed by 55 cycles each consisting of 95 °C for 10 s, 50 °C for 5 s, 72 °C for 8 s; and a final hold at 95 °C for 10 s, 65 °C for 60 s, and 97 °C for 1 s. The matrix gene primers are presented in Table 3.

### 4.7. Classical PCR and Sanger Sequencing

Full-length HA, NA, NS1, and PB1 genes were amplified with Phusion high-fidelity DNA polymerase (New England Biolabs, Ipswich, MA, USA), using M13-tagged primers [63]. The thermal cycling conditions were: 98 °C for 30 s; followed by 30 cycles consisting of 98 °C for 30 s, the respective annealing temperature for 45 s, 72 °C for 3 min; and a final hold at 72 °C for 5 min. PCR products were electrophoresed on a 1% agarose gel, and target bands were extracted using the QIAquick gel extraction kit (Qiagen). Purified amplicons were subjected to Sanger sequencing using universal M13F and M13R primers (as shown in Table 3).

### 4.8. Viral Gene Sequence Construction and Quasispecies Analysis

Full-length HA, NS1, and other viral gene sequences from each passage were constructed, and pairwise alignment was carried out against the P0 sequence to screen for any mutations. Sequencing chromatograms were also analyzed for the presence of viral RNA quasispecies, denoted by base positions with double peaks. Sub-dominant peaks were considered as quasispecies if they fulfilled the following criteria: the base position had a quality score of ≤45; the sub-dominant peak was aligned with and had a height of at least 25% or greater compared to the dominant peak. All chromatograms were visualized using FinchTV software (Version 1.4.0).

### 4.9. Microneutralization Assays

These assays were carried out to determine any differences in the titers and profiles of neutralizing antibodies against parental (P0) and passaged (P5) viral populations. A total of 20 serum samples from known influenza-antibody-positive healthcare workers [64] were tested. MDCK-SIAT1 cells (5 × 10^5^ per well) were initially seeded in 96-well plates and allowed to grow overnight. Two-fold serial dilutions of each serum sample were performed from a starting dilution of 1:20 until 1:2560. Each serum dilution was mixed with an equal volume (100 µL) of virus containing 5 × 10^3^ PFU of P0 or P5 virus. Each virus–serum mixture was incubated at 35 °C for 2 h before the mixture (50 µL) was used to infect MDCK-SIAT1 monolayers in DMEM supplemented with 1× antibiotic–antimycotic and 3 µg/mL TPCK-trypsin. All infections were carried out in duplicate, and cells were then incubated at 35 °C for up to 72 h. Virus-only controls, serum-only controls, and mock-infected controls were also included. Cells were observed for the presence of cytopathic effects (CPEs) at the end of the incubation period. The highest dilution at which a complete monolayer was maintained (no CPE observed) was determined as the antibody titer.

### 4.10. Homology Modelling of Mutant HA and PB1 Proteins

The published experimental structures of influenza H3N2 HA and PB1 protein were downloaded from the Protein Data Bank (PDB). Specifically, chain B of the crystal structure of A/Brisbane/10/2007(H3N2) influenza virus hemagglutinin in apo form [34] was retrieved as the reference structure for the HA protein (PDB code: 6AOR). Chain B of the crystal structure of A/Northern Territory/60/1968(H3N2) influenza virus RNA-directed RNA polymerase catalytic subunit in apo form [35] was also retrieved as the template for the PB1 protein (PDB code: 6QNW). Accordingly, Modeller software (version 10.2) was used for homology modelling of the HA protein harboring the D112G mutation and the PB1 protein with the Q84H, G101C, and R316Q mutations [65].

## 5. Conclusions

In summary, this in vitro study provided evidence that MDCK-SIAT1 cells are more effective than CEF cells in supporting the propagation of a clinical isolate of H3N2, as demonstrated by increased viral titers. This finding supports the use of mammalian cells as an alternative to embryonated hens’ eggs to produce influenza vaccines. Furthermore, serial passaging of the A/Singapore/G2-31.1/2014 strain in MDCK-SIAT1 cells resulted in multiple influenza virus mutations associated with larger virus plaques.

A limitation of this study is that only four genes of a single influenza virus strain were sequenced for mutational analysis. Further studies should include the whole-genome analyses of more H3N2 strains isolated across different years and regions to provide a more comprehensive perspective of the evolutionary patterns of H3N2. Future investigations should also compare virus passaging in embryonated eggs versus CEF cultures. Finally, in vivo studies would also be warranted to determine the functional significance of specific H3N2 mutations and to evaluate their contributions to influenza virulence. In the quest for more effective vaccines, these methods constitute a multi-faceted strategy to address the challenges of H3N2 vaccine mismatch and egg adaptation.

## Figures and Tables

**Table 1:** Summary of specific mutations in A/Singapore/G2-31/2014 following five serial passages in MDCK-SIAT1 and chick embryo fibroblast (CEF) cells. The dash symbol (–) indicates absence of specific mutations. N.D. denotes the passages in which the full-length gene could not be amplified.

	HA		NS1		NA		PB1	
Virus Passage	MDCK-SIAT1	CEF	MDCK-SIAT1	CEF	MDCK-SIAT1	CEF	MDCK-SIAT1	CEF
1	–	–	–	–	–	N.D.	–	N.D.
2	–	N.D.	–	N.D.	–	N.D.	–	N.D.
3	–	N.D.	–	N.D.	A736G (silent)	N.D.	A275C (Q84P)	N.D.
							G325T (G101C)	
4	–	N.D.	–	N.D.	–	N.D.	–	N.D.
5	A1399G (D457G)	N.D.	–	N.D.	–	N.D.	–	N.D.

**Table 2:** Comparison of titers of neutralizing antibodies against MDCK-SIAT1 passage P0 and P5 viruses in a panel of influenza-immune sera from 20 subjects.

Serum Code	Neutralizing Antibody Titers against P0 and P5 Viruses	
	P0	P5
FMT167	160	160
FMT486	160	160
FMK138	160	160
FMK025	160	160
FMK041	160	160
FMT018	160	160
FMK123	320	320
FMT034	160	320
FMT048	160	320
FMT052	320	320
FMT071	160	320
FMT108	160	160
FMT163	160	320
FMT182	320	320
FMT190	320	320
FMT239	320	320
FMT416	160	160
FMT448	160	160
FMT468	160	160
FMK013	160	160

**Table 3:** List of PCR primers (and their sequences) used in this study.

Code Name	Primer Sequence (5′-3′)	Purpose
HA1F	AGCAGGGGATATTTTTATTAACC	Forward primer for preliminary HA1 screening
HA1R	CAACCATCCACCATTCCCTC	Reverse primer for preliminary HA1 screening
M1-M2F	CATCCTGTTGTATATGAGGCCCAT	Forward primer for real-time qPCR
M1-M2R	GGACTGCAGCGTTAGACGCTT	Reverse primer for real-time qPCR
NS1-M13F	TGTAAAACGACGGCCAGTAGCAAAAGCAGGGTGACAAAGACA	Forward primer for NS1 amplification
NS1-M13R	CAGGAAACAGCTATGACCAGTAGAAACAAGGGTGTTTTTTAT	Reverse primer for NS1 amplification
HA-F1-M13F	TGTAAAACGACGGCCAGTAAAGCAGGGGATAATTCTA	Forward primer for HA Fragment 1
HA-F1-M13R	CAGGAAACAGCTATGACCATTGCTGCTTGAGTGCTT	Reverse primer for HA Fragment 1
HA-F2-M13F	TGTAAAACGACGGCCAGTGGTTACTTCAAAATAC	Forward primer for HA Fragment 2
HA-F2-M13R	CAGGAAACAGCTATGACCAGTAGAAACAAGGGTGTTTT	Reverse primer for HA Fragment 2
NA-F1-M13F	TGTAAAACGACGGCCAGTAGCAAAAGCAGGAGT	Forward primer for NA Fragment 1
NA-F1-M13R	CAGGAAACAGCTATGACCCGACATGCTGAGCACTYCCTGAC	Reverse primer for NA Fragment 1
NA-F2-M13F	TGTAAAACGACGGCCAGTGAACTTGTRCAGTRGTAATG	Forward primer for NA Fragment 2
NA-F2-M13R	CAGGAAACAGCTATGACCAGTAGAAACAAGGAG	Reverse primer for NA Fragment 2
PB1-F1-M13F	TGTAAAACGACGGCCAGTAGCRAAAGCAGGCAAACCAT	Forward primer for PB1 Fragment 1
PB1-F1-M13R	CAGGAAACAGCTATGACCTGTTCAAGCTTTTCRCAWATGC	Reverse primer for PB1 Fragment 1
PB1-F2-M13F	TGTAAAACGACGGCCAGTCRATGACCAAAGATGCWGA	Forward primer for PB1 Fragment 2
PB1-F2-M13R	CAGGAAACAGCTATGACCAAGGTCATTGTTTATCATRTTG	Reverse primer for PB1 Fragment 2
PB1-F3-M13F	TGTAAAACGACGGCCAGTGTGGCYAATTTYAGCATGGAG	Forward primer for PB1 Fragment 3
PB1-F3-M13R	CAGGAAACAGCTATGACCAGTAGAAACAAGGCATTT	Reverse primer for PB1 Fragment 3

## Data Availability

Not applicable.

## Supplementary Materials

The following supporting information can be downloaded at: https://www.mdpi.com/article/10.3390/ijms232012408/s1.
